# Value of MRI to Improve Deep Learning Model That Identifies High-Grade Prostate Cancer. Comment on Gentile et al. Optimized Identification of High-Grade Prostate Cancer by Combining Different PSA Molecular Forms and PSA Density in a Deep Learning Model. *Diagnostics* 2021, *11*, 335

**DOI:** 10.3390/diagnostics11071213

**Published:** 2021-07-05

**Authors:** Joshua S. Jue, David Mikhail, Javier González, Mahmoud Alameddine

**Affiliations:** 1 Department of Urology, Lenox Hill Hospital, Northwell Health, Zucker School of Medicine at Hofstra/Northwell, New York, NY 10028, USA; DMikhail@northwell.edu; 2 Department of Urology, Hospital General Universitario Gregorio Marañón, 28007 Madrid, Spain; fjgg1975@yahoo.com; 3 Ottumwa Regional Health Center, Department of Urology, Ottumwa, IA 52501, USA; dralameddine@hotmail.com

Prostate-specific antigen (PSA) has been criticized for its low specificity for prostate cancer, which has led to the increased adoption of additional biomarkers, PSA density (PSAD), and multiparametric magnetic resonance imaging (mpMRI) to increase the localization, risk stratification, and diagnosis of prostate cancer. In this prospective study, the authors found that a deep learning approach may aid in the diagnosis of aggressive forms of prostate cancer, with an accuracy of 55% and precision of 93% using total PSA (tPSA), free PSA (fPSA), isoform-2 of proPSA (p2PSA), and PSAD [1]. The use of a deep learning approach is interesting, and may become more widely adopted in the future as new technology, algorithms, and models are becoming increasingly used in medicine. For now, while costs remain a concern within medicine, a further stratification of patients by PSA value may increase the accuracy and precision of PSAD in this deep learning approach. Using prospective, multi-institutional trial data from the 4Kscore, PSAD predicted clinically significant prostate cancer best for increasing values of PSA [2]. The area under the receiver operating characteristic curve (AUC) of PSAD was significantly greater than PSA in patients with significant prostate cancer in the PSA range of 4–10 ng/mL (AUC: 0.72 vs. 0.57, *p* < 0.0001) and PSA > 10 ng/mL (AUC: 0.82 vs. 0.68, *p* < 0.0001), but not PSA < 4 ng/mL (*p* = 0.23) [2]. The stratification of patients by PSA within a deep learning model may be a simple adjustment that can improve its prediction of significant prostate cancer, as it has with PSAD.

Level 1 evidence has established prostate mpMRI as an essential instrument in the diagnosis, treatment, and surveillance of localized prostate cancer [3]. MRI visible lesions have also been shown to harbor the most clinically significant cancer within the prostate gland [4]. Unfortunately, this study did not utilize mpMRI and solely performed a 16-core template biopsy instead of an MRI/ultrasound fusion biopsy. PSAD and MRI visibility have been used in conjunction to enhance the care of an active surveillance cohort. One study utilized a linear mixed-effects longitudinal model with random intercepts for patients and a random non-linear tie effect using natural cubic splines to logarithmically describe PSAD over time [5]. In the model, Gleason-grade and MRI visibility were significant predictors of event-free survival [5]. PSAD may have non-linear relationships over time that can further complicate diagnostic tools. Deep learning models may become increasingly helpful in the future diagnosis of high-grade prostate cancer, with particular emphasis on PSAD and MRI visibility.

## Data Availability

Not applicable.
